# Antifungal Activity of *Lactobacillus plantarum* ZZUA493 and Its Application to Extend the Shelf Life of Chinese Steamed Buns

**DOI:** 10.3390/foods11020195

**Published:** 2022-01-12

**Authors:** Shanshan Zhao, Xiangmei Hao, Fengyuan Yang, Yuan Wang, Xiaomiao Fan, Yanping Wang

**Affiliations:** 1 Henan Key Laboratory of Ion Beam Bio-Engineering, College of Physics, Zhengzhou University, Zhengzhou 450000, China; zsszd@gs.zzu.edu.cn (S.Z.); hxm_dy@163.com (X.H.); yangfy@gs.zzu.edu.cn (F.Y.); wangyuany5@163.com (Y.W.); fanxiaomiao1125@163.com (X.F.); 2 Henan Key Laboratory of Ion Beam Bio-Engineering, School of Agricultural Science, Zhengzhou University, Zhengzhou 450000, China

**Keywords:** lactic acid bacteria, fungi, antifungal activity, Chinese steamed buns

## Abstract

Lactic acid bacteria (LAB) can produce many kinds of antifungal substances, which have been widely proven to have antifungal activity. In this study, 359 strains of LAB were screened for antifungal activity against *Aspergillus niger (A. niger)* using the 96-well microtiter plate method, and three showed strong activity. Of these, ZZUA493 showed a broad-spectrum antifungal ability against *A. niger*, *Aspergillus oryzae*, *Trichoderma longibrachiatum, Aspergillus flavus* and *Fusarium graminearum*. ZZUA493 was identified as *Lactobacillus plantarum*. Protease treatment, the removal of hydrogen peroxide with catalase and heat treatment had no effect on the antifungal activity of the cell-free supernatant (CFS) of ZZUA493; organic acids produced by ZZUA493 appeared to have an important role in fungal growth inhibition. The contents of lactic acid, acetic acid and phenyllactic acid in the CFS tended to be stable at 48 h, and amounted to 28.5, 15.5 and 0.075 mg/mL, respectively. In addition, adding ZZUA493, as an ingredient during their preparation, prolonged the shelf life of Chinese steamed buns. Overall, ZZUA493 appears to have good potential as a fungal inhibitor for food preservation.

## 1. Introduction

Filamentous fungi such as Aspergilli and Fusaria are major causes of spoilage in foods, crops and livestock forage [1]. Food corruption caused by mold can cause huge economic losses, and the toxin produced by mold will endanger people’s health and safety [2]. For the food industry, food safety and hygiene are some of the key issues in its development process. Fungal contamination, which causes the physical and chemical deterioration of food, is often accompanied by detrimental sensory changes, including changes in taste, odor, color and texture, in meat, seafood, dairy products and grains [3]. Many physical or chemical approaches have been taken and implemented to control the growth of filamentous fungi. For example, pulsed light treatment could inactivate *Penicillium expansum* inoculated in apple juice [4]; the efficacy of UV-C light-emitting diodes and a low-pressure mercury lamp to inactivate *Penicillium expansum* spores on apple skin was assessed [5]; chlorine dioxide (ClO_2_) fumigation inhibited *Penicillium expansum* growth and patulin production [6]; natamycin application had a strong inhibitory effect on *Botrytis cinerea* and *Penicillium expansum* [7]. However, these approaches are not considered the best solution due to the problems of cost and side effects. In recent years, there has been increased concern about the microbiological safety of various foods. Biological preservatives have become a new favorite in the international food additive market because of their natural origins, high efficiency, non-toxicity or low toxicity and lack of damage to the original flavor of food. *Bacillus subtilis* and *Bacillus amyloliquefaciens* had significant inhibitory effects on *Aspergillus flavus* and aflatoxin [8]. Sadiq et al. reviewed the research progress of lactic acid bacteria (LAB) as antifungal and antimycotoxin agents [9]. Rodriguez Assaf et al. isolated several fungi, including *Aureobasidium pullulans*, *Cryptococcus magnus*, *Metschnikowia pulcherrima* and *Rhodotorula glutinis*, with antifungal activity from fermentation microenvironments and the surfaces of refrigerated grapes [10]. Recent trends in detecting, controlling and detoxifying the patulin mycotoxin were observed using biotechnology methods [11].

LAB, as probiotics, are generally recognized as safe (GRAS) strains [12]. It is widely known that the acidic substances produced by LAB fermentation reduce the pH value of the environment and cause intracellular acidic stress, resulting in unfavorable conditions for the growth of fungus in food products [13]. These acidic substances, including lactic acid (LA), acetic acid (AA), propionic acid, phenyllactic acid (PLA) and fatty acid, have been proven to have an antifungal ability [14]. In addition to organic acids, some peptides and esters [15] have also been identified as antifungal compounds. LAB are naturally present in many foods and widely used as bio-preservatives because of their antifungal properties. Lan et al. sprayed a certain concentration of LAB fermentation broth on the surfaces of grapes, which could prolong the shelf life of grapes containing *Penicillium oxalate* for 6 days [16]. Muhialdin et al. reported that the supernatant of the four strains of LAB with antifungal activity that were screened from food could prolong the shelf life of cheese by 19–29 days at 4 °C and 6–12 days at 20–30 °C [17]. Alireza Sadeghi et al. found that the growth of *A. niger* on produced bread was also remarkably decreased using controlled sourdough (containing the selected LAB isolate) in comparison with spontaneous sourdough and control bread [18]. Chinese steamed buns (CSB) are a food made from wheat flour and water as raw materials and yeast as the main starter, kneaded into dough, fermented, shaped and steamed after awakening. As the traditional staple food in China, CSB occupy an important position in the population’s diet structure. With the acceleration in the pace of the population’s lives, the industrialization of staple foods will definitely become a future development trend, and the industrialization of CSB will be an important part of this [19]. However, because CSB are rich in nutrition and have a high water content, they can be easily corrupted by bacteria and mold during storage, and the shelf life is very short; all these factors restrict the development pace of the industrial production of steamed bread [20]. Therefore, screening and identifying the LAB with a high organic acid yield and strong antifungal ability can provide potential biological control agents for prolonging CSB’s shelf life.

The objectives of this study were to screen for LAB strains with antifungal activity, and to investigate the antifungal activity and properties of antifungal substances of the selected strain. In addition, the selected strain was applied to CSB to evaluate its effect on shelf life extension.

## 2. Materials and Methods

### 2.1. Strains and Growth Conditions

A total of 359 strains were isolated from alfalfa samples in our laboratory, which were characterized as LAB based on physiological and biochemical experiments [21]. They were cultured on de Man–Rogosa–Sharpe (MRS) agar (Beijing Aoboxing Biotechnology Co., Ltd., Beijing, China) at 30 °C for 48 h in an anaerobic environment.

Genomic DNA of fungi (4 strains) were extracted by CTAB (cetyltrimethylammonium ammonium bromide) cleavage, and amplified by the universal primers of fungal ITS (primer sequence ITS1: 5 ′-TCCGTAGGTGaACCTGCGG-3′, ITS4:5 ′-TCCTCCGCTTATTGATATG-3′); the reaction system was 30 uL: Buffer 3 μL, dNTP 2 μL, upstream and downstream primers 3 μL, respectively, DNA template 1 μL, ddH_2_O 17.8 μL, Q5 DNA Polymerase 0.2 μL. The reaction conditions were 95 °C for 5 min, 95 °C, 30 s, 55 °C, 30 s, 72 °C, 1 min, 35 cycles; finally, 72 °C, 10 min. PCR products were detected with 1% agarose gel, and the target band was cut and recycled and sent to BGI (Beijing Genomics Institute, Beijing, China) for sequencing. The BLAST results were compared with the genomic data available on National Center for Biotechnology Information (NCBI) and fungi (4 strains) were identified as *Aspergillus niger* (*A. niger*), which was obtained from alfalfa, *Aspergillus oryzae* (*A. oryzae*) and *Trichoderma longibrachiatum* (*T. longibrachiatum*), which were obtained from CSB, and *Aspergillus flavus* (*A. flavus*), which was obtained from oranges (Figure S1). *Fusarium graminearum* (*F. graminearum*) was acquired from the Institute of Agricultural Products Processing, Chinese Academy of Agricultural Sciences, and was obtained from cereal. Putrefactive bacteria (*Staphylococcus aureus* ATCC 29213, *Micrococcus luteus* ATCC 4698, *Bacillus subtilis* ATCC 6633, *Pseudomonas aeruginosa* ATCC 27853, *Escherichia coli* ATCC 30105, *Listeria monocytogenes* BAA) were used as indicator bacteria. The fungal and bacterial strains were inoculated on Potato Dextrose Agar (PDA) (potato extract, 3.0 g; glucose, 20.0 g; agar, 14.0 g/L) and Luria–Bertani (LB) agar (yeast extract, 5.0 g; peptone, 10.0 g; sodium chloride, 5.0 g; agar, 15.0 g/L), incubated at 30 °C for 72 and 48 h, respectively.

### 2.2. Preparation of Fungal Spore Suspensions

The concentrations of fungal spore suspensions were adjusted to approximately 1.42 × 10^7^ cfu mL^−1^ with saline (9 g/L NaCl solution), to achieve an optical density (OD) of 0.6 at 600 nm (OD_600_). The prepared spore suspensions of fungi were diluted 0, 4, 16 and 32 times, for experiments.

### 2.3. Preparation of LAB Fermentation Cell-Free Supernatant (CFS)

After incubation at 30 °C for 48 h in MRS broth, the CFS of LAB strains was obtained by centrifugation (8000× *g* for 10 min at 4 °C), part of which was freeze-dried and re-dissolved to a 10-fold higher concentration (10 × CFS).

### 2.4. Screening of LAB Strains for Antifungal Properties In Vitro

#### 2.4.1. Preliminary Screening

Microtiter plates (96-well) were used for preliminary screening, as described previously [22], with some modifications. A suspension of spores of *A. niger* (100 μL) and LAB CFS (100 μL) or MRS broth (100 μL, as a control) that were 4 or 16 times diluted or undiluted was added to sterile 96-well microtiter plates, and then incubated at 30 °C for 48 h. The absorbance value (OD_600_) at 600 nm was measured using an enzyme labeling instrument, and the antifungal rate was calculated using Formula (1), which is as follows:

$$Antifungal\;rate\;\left(\%\right)=\left(1-\frac{OD_{LAB}}{OD_{CONTROL}\;}\right)\times 100$$

where OD_LAB_ represents the OD_600_ of mold spores cultured in LAB fermentation supernatant for 48 h; OD_control_ indicates the OD_600_ of mold spores cultured in MRS liquid for 48 h.

#### 2.4.2. Rescreening

The dual-culture overlay assay [23] was used for the rescreening, with some modifications. Two lines, approximately 2.5 mm long, were drawn on MRS agar with LAB strains, and then the plates were incubated in an anaerobic environment for 24 and 48 h. The upper layer of agar was poured (5 mL/plate of PDA, containing the 32-times diluted suspension of spores of 4 kinds of fungi, respectively). After solidification, the plates were incubated at 30 °C, and the antifungal activity was observed visually, after 24 and 48 h.

### 2.5. Identification of ZZUA493 Strain

#### 2.5.1. Morphological, Physiological and Biochemical Tests of LAB

Morphology and Gram-staining response were examined after 24 h of incubation on MRS agar. Catalase activity and gas production from glucose were determined. Growth at different temperatures was observed in MRS broth after incubation at 5 and 10 °C for 14 d, respectively, and at 30, 40, 45 and 50 °C for 7 d. Salt tolerance was determined in MRS broth with NaCl at 3.0 and 6.5% for 2 d. Growth of LAB at different pH—3.0, 3.5, 4.0, 4.5, 5.0, 5.5, 6.0, 7.0, 8.0, 8.5, 9.0, 9.5 and 10.0—was determined in MRS broth after incubation at 30 °C for 7 d [21].

#### 2.5.2. Drawing of Growth Curve of LAB

The LAB were cultured in a scribed line; a single colony was selected and added to a triangular flask containing 100 mL of MRS liquid, placed in a 30 °C constant temperature incubator, the bacterial suspension was taken every 2 h, and the OD_600_ and pH values were measured.

#### 2.5.3. 16S rRNA Gene Sequencing

The DNA of ZZUA493 was extracted using a Bacteria Gen DNA extraction kit (Jiangsu Cowin Biotech Co.,Ltd., Taizhou, Jiangsu, China), and then it was subjected to a specific polymerase chain reaction (PCR). The sequence of the primer was 27F: AGAGTTTGATCCTGGCTCAG and 1492R: GGYTACCTTGTTACGACTT (5′–3′). PCR reaction system (2 * Taq MasterMix; forward primer, 10 M; reverse primer, 10 M; template DNA; RNase-free water) and reaction conditions (94 °C 2 min; 94 °C 30 s; 55–65 °C 30 s; 72 °C 30 s; 72 °C 2 min; 35 cycles) were followed, according to the manufacturer’s instructions. After agarose gel electrophoresis (TAE, 1% *w*/*v*), the 16Sr DNA was sequenced by BGI (Beijing Genomics Institute, Beijing, China). In order to determine the genus of the strain selected, sequence splicing was performed by Seqman splicing software, and then the BLAST results were compared with the genomic data available on NCBI.

### 2.6. Studies on the Antifungal Activity and Properties of Antifungal Substances of ZZUA493

#### 2.6.1. Antifungal Activity of ZZUA493 CFS

A study on the antifungal activity of CFS was performed as described previously [24]. The CFS of ZZUA493, or 10 × CFS of ZZUA493, or 10 × CFS of ZZUA493 with the pH adjusted to 6.0, were added at a concentration of 5% to liquid PDA agar, mixed well and poured into the plates. The CFS and 10 × CFS were replaced with an equivalent volume of MRS to form the control check (CK). After solidification, *F. graminearum* or *A. niger* was inoculated onto the plates, and then incubated at 30 °C. The antifungal activity was observed after 3 d. 

#### 2.6.2. Protease Treatment

The pH of CFS was adjusted to the optimum value of each enzyme (trypsin, pepsin and proteinase K). After incubation at 37 °C for 2 h, 2 mg/mL of each proteolytic enzyme of CFS was boiled for 5 min to inactivate the enzymes. After cooling, the pH of each treatment group was adjusted to the original value (3.8). The CK contained MRS broth with enzymes. Treated CFS or MRS broth (100 μL) and fungal spore suspension (100 μL) were placed into 96-well plates, and then incubated at 30 °C for 48 h. The absorbance (OD_600_) was measured by a microplate reader, and the inhibition rate was calculated.

#### 2.6.3. Hydrogen Peroxide Removal

Catalase (Mettler-Toledo AG, Greifensee, Switzerland) was used to remove any hydrogen peroxide in the CFS. The procedure was as for the protease treatment above.

#### 2.6.4. Acid Exclusion

In order to test the effect of pH on the antifungal activity, the pH of CFS was adjusted to 4.5–6.5 with 1 M NaOH solution. Treated CFS or MRS broth (100 μL) and fungal spore suspension (100 μL) were placed into 96-well plates, and then incubated at 30 °C for 48 h. The absorbance (OD_600_) was measured using a microplate reader, and the inhibition rate was calculated.

#### 2.6.5. Heat Treatment

The CFS was placed in water baths at 60, 80 or 100 °C for 2 h. Treated CFS or MRS broth (100 μL) and fungal spore suspension (100 μL) were placed into 96-well plates, and then incubated at 30 °C for 48 h. The absorbance (OD_600_) was measured using a microplate reader, and the inhibition rate was calculated. 

#### 2.6.6. Determination of Organic Acid Content of ZZUA493 CFS

LA, AA and PLA in CFS were determined using High-Performance Liquid Chromatography (HPLC), as described previously [25]. After centrifugation (8000× *g* for 10 min at 4 °C), the CFS was sterilized with a micro filter (0.22 μm, JIN TENG) and directly injected into the HPLC system (Waters, Milford, MA, USA), fitted with a Symmetry C18 column (250 × 4.6 mm, 5 μm particle size; Waters). The mobile phase was 0.05% aqueous TFA (solvent A) and methanol with 0.05% TFA (solvent B). The linear gradient was as follows: 0–20 min—10–100% B; 20–30 min—100% B. The flow rate was set to 0.6 mL/min, column temperature to 55 °C and a volume of 10 μL was injected. Organic acids were detected at a UV wavelength of 210 nm and quantified by comparison with external standard solutions.

Treated CFS or LA with concentrations of 30 mg/mL or AA with concentrations of 15 mg/mL or PLA with concentrations of 0.075 mg/mL or a mixture of LA, AA and PLA in the same concentration as CFS or MRS broth (100 μL) and fungal spore suspension (100 μL) were placed into 96-well plates, and then incubated at 30 °C for 48 h. The absorbance (OD_600_) was measured by a microplate reader, and the inhibition rate was calculated.

### 2.7. Cloning of Genes Associated with the Related of Bacteriocin of ZZUA493

Rapid PCR amplification: The reported genes encoding bacterin synthesis from *Lactobacillus plantarum* C11, NC8, V90, J23, J51, WCFS1 and 423 were found from the NCBI database, and specific primers were designed [26] (Table S1). In addition, three other specific primers (Table S1) were designed to validate the presence of pediocin, enterocin and plantaricin for PCR amplification verification [27]. PCR system of each gene: 10 μmol/L upstream and downstream primers of 2 μL for each gene; 1 μL of a DNA template; 25 μL of a Taq enzyme; ddH_2_O was added to make up 50 μL. Reaction conditions: pre-denaturation at 94 °C for 3 min. There were 30 cycles of denaturation at 94 °C for 30 s, annealing at 50–60 °C for 30 s, extension at 72 °C for 40 s and extension at 72 °C for 10 min. After PCR amplification, 5 μL of PCR products was subjected to agarose gel electrophoresis (1.5% agarose, 1 × TAE, 150 V) for 20 min. A 2000 bp molecular size standard ladder was used. The gel was stained with ethidium bromide and analyzed using a Gel Doc TM XR system (Bio-Rad Co., Ltd., Hercules, CA, USA).

### 2.8. Testing of ZZUA493 for Extending the Shelf-Life of CSB 

ZZUA493 was cultured in MRS broth at 30 °C for 12 h, and then the biomass was collected by cold centrifugation. After pouring off the supernatant, the thalli were washed and resuspended in sterile water to obtain a cell suspension. The suspension was adjusted to an OD_600_ of 0.5 (2.31 × 10^8^ cfu mL^−1^) before use. To produce sourdough, the cell suspension (2 mL) was immediately mixed with wheat flour (100 g; from a local supermarket) and water (100 mL) and incubated at 30 °C for 14 h. The cell suspension was replaced with sterile water in the CK.

**Figure 1:**
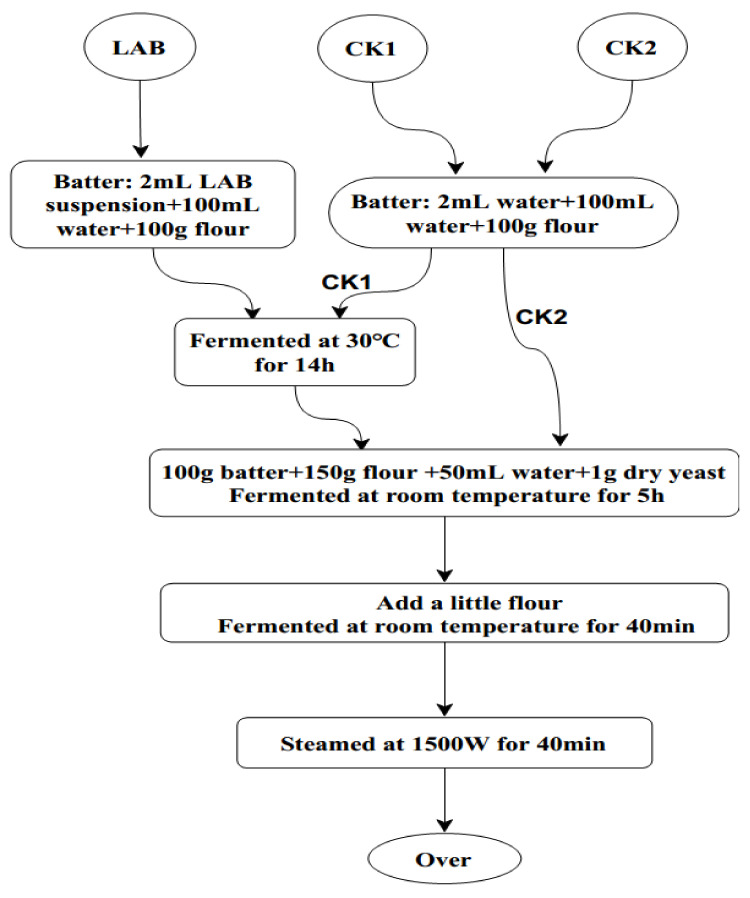
Steps to produce CSB with or without LAB. CSB, Chinese steamed buns; LAB, lactic acid bacteria, Batter: 2 mL ZZUA493 suspension + 100 mL water + 100 g flour fermented at 30 °C for 14 h; CK1, Batter: 2 mL water + 100 mL water + 100 g flour fermented at 30 °C for 14 h control; CK2, Batter: 2 mL water + 100 mL water + 100 g flour unfermented control.

The production process is shown in Figure 1. CSB were produced from a mixture of 150 g of flour, 100 g of sourdough, 1 g of instant dry yeast (Angel Yeast(Yili)Co.,Ltd., Yili, Xinjiang, China) and 50 mL of water. After stirring, the dough was incubated at room temperature (approximately 22 °C) for 5 h. The dough was smoothed with a small amount of flour, and then divided into pieces of 50–55 g. The dough pieces were rolled into balls, incubated in a steamer at room temperature for 30–40 min and then steamed at 100 °C for 30–35 min. The steamed bread was placed in a clean environment for 2 h and aired to room temperature. The steamed bread samples of the experimental group and the two control groups were sliced, placed into a sterilized and dry plate, and part of it was placed in a clean edible bag. Three parallel procedures were set in each group, and the time was recorded. Photos were taken every day to observe the gap between the experimental group and the control group.

### 2.9. Statistical Analysis

Biomass was calculated as the number of colony-forming units per mL of sample (cfu mL^−^^1^). All the experiments were conducted in triplicate. Data obtained from microbiology were analyzed using ANOVA and displayed using Origin drawing software. The results were recognized as statistically significant at *p* < 0.05.

## 3. Results and Discussion

### 3.1. Screening of LAB Strains for Antifungal Activity

**Figure 2:**
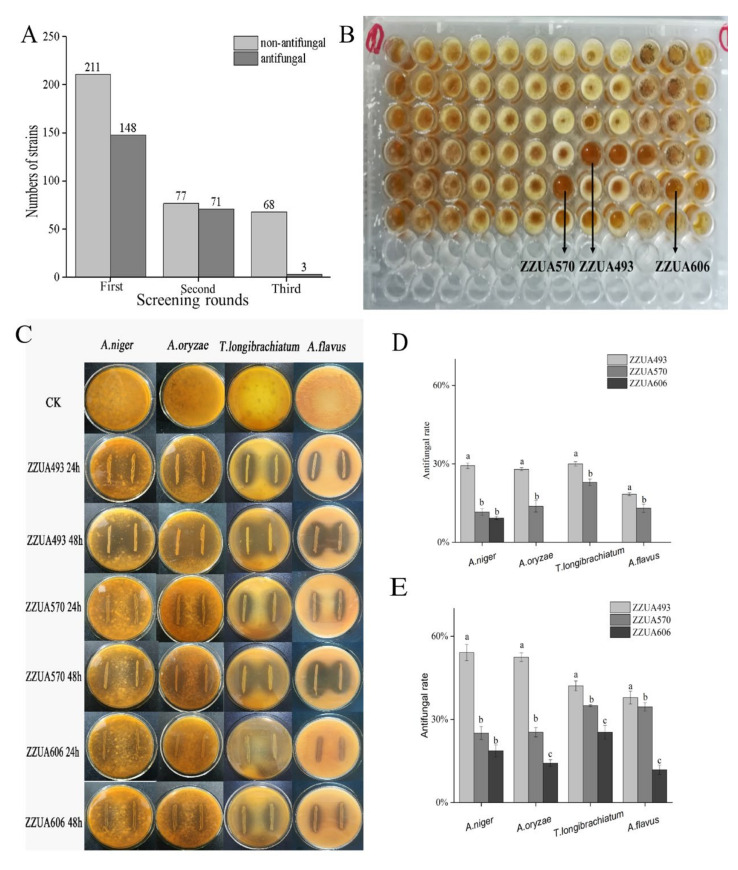
Detection of antifungal ability by 96-well microtiter plate and dual-culture overlay assay method. The strains with antifungal ability against *A. niger* screened out in three rounds by 96-well microtiter plate method (**A**); 96-well plate method was used to detect the antibacterial activity of 71 LAB strains against *A. niger* (**B**); The antifungal activity of ZZUA493, ZZUA570 and ZZUA606 against 4 kinds of fungi (*A. niger*, *A. oryzae*, *T. longibrachiatum* and *A. flavus*), tested by dual-culture overlay assay method (**C**); Antifungal rate of ZZUA493, ZZUA570 and ZZUA606 against 4 kinds of fungi after 24 h incubation (**D**); Antifungal rate of ZZUA493, ZZUA570 and ZZUA606 against 4 kinds of fungi after 48 h incubation (**E**). Values with different superscript lowercase letters show significant differences among treatments of ZZUA493, ZZUA570 and ZZUA606 (*p* < 0.05). CK, no LAB control; *A. niger, Aspergillus niger; A. oryzae, Aspergillus oryzae; T. longibrachiatum, Trichoderma longibrachiatum; A. flavus, Aspergillus flavus*.

The antifungal activity of LAB was screened from 359 strains using the 96-well microtiter plate method. Among them, 148 strains had antifungal activity against the spore suspension diluted 16 times (8.88 × 10^5^ cfu mL^−1^). At the second round, 71 of the 148 strains were selected for the antifungal activity against the spore suspension diluted four times (3.55 × 10^6^ cfu mL^−1^). In the third round (Figure 2A), three strains (ZZUA493, ZZUA570 and ZZUA606) had antifungal activity against the undiluted spore suspension (1.42 × 10^7^ cfu mL^−1^) (Figure 2B). In this study, screening with microtiter plates was found to be simple and convenient for massive screening, while the dual-culture overlay assay could accurately compare the relative activities of ZZUA493, ZZUA570 and ZZUA606. To characterize the antifungal activity of the three best LAB strains in more detail, the dual-culture overlay assay was used with *A. niger*, *A. oryzae, T. longibrachiatum* and *A. flavus* (Figure 2C); all three LAB strains exhibited clear regions indicating an inhibition of fungal growth, with ZZUA493 having the largest clear regions and ZZUA606 the smallest. The inhibition rate of three strains of LAB on four fungi is shown in Figure 2D,E. The antibacterial activity of ZZUA493 on four fungi was significantly higher than that of ZZUA570 and ZZUA606 (*p* < 0.05). Therefore, ZZUA493 had the strongest antifungal activity and was selected for further experiments. Moreover, the selection of fungal targets is also important because they should represent the occurrence of pollutants and biodiversity [28]. In this study, four fungal targets were selected from contaminated products; of these, *A. niger* was the most sensitive, and *A. flavus* was the least sensitive to the antifungal activity of LAB strains; therefore, *A. niger* was selected as the fungal target for the subsequent experiments. 

### 3.2. Morphological, Physiological and Biochemical Properties and Molecular Identification of ZZUA493

**Figure 3:**
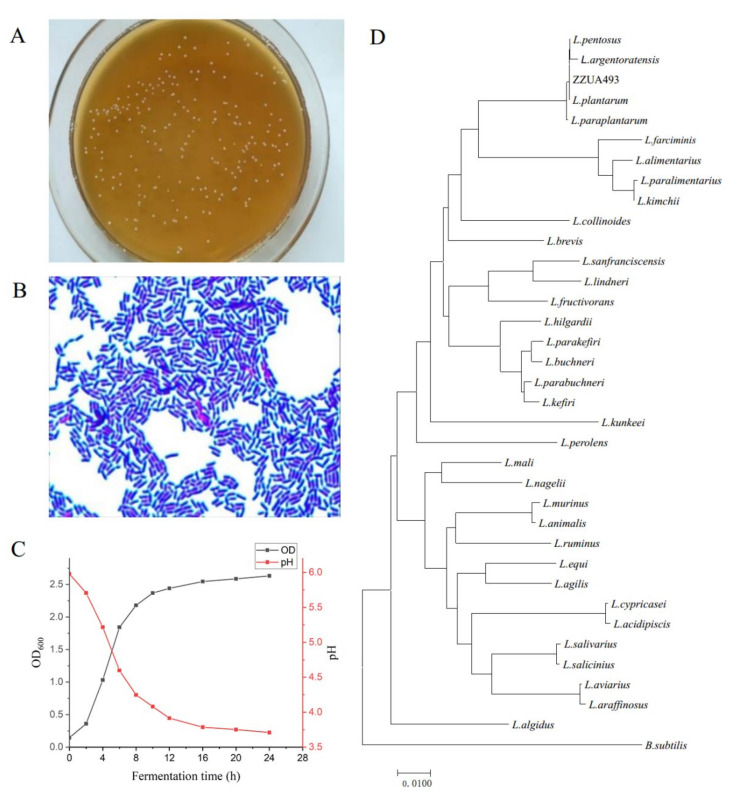
Identification of ZZUA493 strain. Colony morphology (**A**); gram staining (**B**); growth and pH change curve of ZZUA493 (**C**); phylogenetic tree obtained from 16Sr RNA gene sequence analysis, giving the genus of ZZUA493; the tree was generated by the neighbor-joining method and the bar shows 1% sequence divergence (**D**).

The physiochemical test showed that ZZUA493 was Gram-positive, catalase-negative, rod-shaped and of homo-type fermentation (Figure 3A,B), which confirmed that it belonged to *Lactobacillus*. The growth of ZZUA493 was not affected under the condition of salt at 3 and 6.5%. In the presence of salt, salt-tolerant microorganisms can reproduce, while other salt-sensitive microorganisms are repressed [29]. In the current study, ZZUA493 could grow in 6.5% salted MRS broth, and this laid the foundation for the application of ZZUA493 in a high-salt environment. After incubation at different temperatures (Table 1), ZZUA493 was weaker at 5 and 50 °C than 10–45 °C, with good heat and cold resistance, which increased the possibility of high-temperature and low-temperature fermentation. Moreover, under the condition of pH 3.0–10.0, the growth was not affected. The growth curve of ZZUA493 was measured within 24 h. As shown in Figure 3C, ZZUA493 was in the logarithmic growth phase at 4–8 h and in the slow growth phase after 12 h. It can be seen that the strain grew rapidly and reached the stable growth phase within 12 h. In addition, at 4–8 h, the pH value of the fermentation broth of the strain declined rapidly, during which organic acids could be produced and accumulated rapidly. The pH changed slowly after 12 h. It is well known that LAB can produce organic acids, and the presence of organic acids will reduce the environmental pH and inhibit the growth of spoilage bacteria. Furthermore, ZZUA493 had strong antibacterial activity on *Staphylococcus aureus*, *Bacillus subtilis*, *Micrococcus luteus*, *Pseudomonas aeruginosa*, *Escherichia coli* and *Listeria monocytogenes* (Table S2). The 16Sr RNA gene sequence analysis (Figure 3D) showed that ZZUA493 belonged to *L. plantarum*, with a homology of 99.93%. The 16Sr RNA gene sequence of strain ZZUA493 has been deposited in the GenBank repository with accession number OM037031.

### 3.3. Studies on Antifungal Activity and Properties of Antifungal Substances of ZZUA493

**Figure 4:**
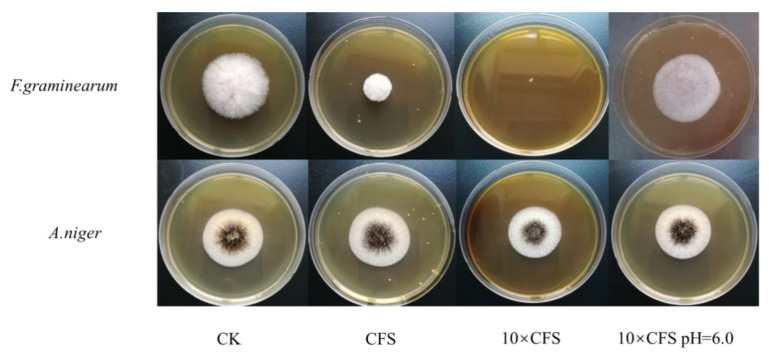
Antifungal effects of the concentrated culture filtrate of ZZUA493 against *F.graminearum* and *A. niger*. *F. graminearum, Fusarium graminearum; A. niger, Aspergillus niger;* CK, no LAB CFS control; CFS, cell-free supernatant; 10 × CFS, 10-fold-concentrated cell-free supernatant; 10 × CFS pH = 6.0, the pH of 10 × CFS was adjusted to 6.0.

In this study, it was found that ZZUA493 had obvious inhibitory regions against *A. niger*, *A. oryzae, T. longibrachiatum* and *A. flavus* using the double-layer plate scribing method (Figure 2C), which indicated that ZZUA493 had broad-spectrum antibacterial activity. The fungal spoilage of grain is often caused by *Fusarium* spp. and results in serious risks to food safety and human health [30]. The high toxicity and carcinogenicity of *Fusarium* spp. mean that inhibiting the growth is critical for effective grain preservation in storage. The addition of the CFS of ZZUA493 showed clear inhibition against *F. graminearum* but low inhibition against *A. niger* (Figure 4), which meant that *F. graminearum* was more sensitive to the CFS. However, the antifungal activity of ZZUA493 against *A. niger* in the previous results was the opposite, which may have been due to the low content of antifungal substances in the 10 × CFS. The metabolites produced by LAB in original and neutralized CFS showed various degrees of antifungal activity. A 10-fold-concentrated culture filtrate of *L. plantarum* completely inhibited *Eurotium repens*, *Eurotium rubrum* and *Penicillium corylophilum* [31]. In this study, *F. graminearum* did not grow at all on the plate containing 10 × CFS. When the pH of the 10 × CFS was adjusted to 6.0, the CFS showed no antifungal activity compared with the CFS that was not neutralized. In a previous report, the antifungal activity of the CFS, after adjustment to pH 7.0, was no better than blank growth medium [32], which is consistent with our results. Therefore, it appeared that either organic acids themselves inhibited the growth of fungi, or the acidic environment produced by the organic acids may be conducive to the antifungal activity of other antifungal substances.

**Figure 5:**
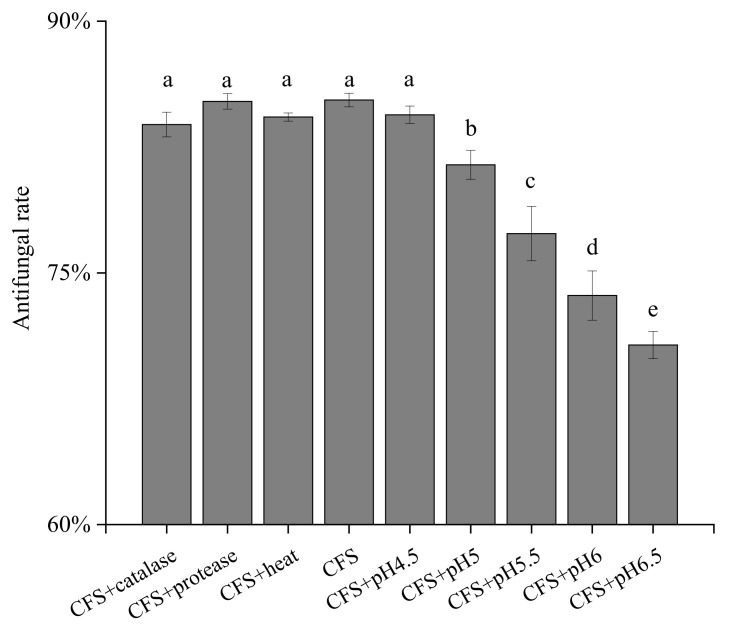
The antifungal rate of CFS after different treatments in 96-well microtiter plate method. Values with different superscript lowercase letters show significant differences among treatments (*p* < 0.05). CFS, cell-free supernatant.

LAB can produce many kinds of antifungal substances in the process of growth, such as organic acids, proteins and peptides. To characterize the antifungal substances of ZZUA493, the CFS was treated with protease enzymes, hydrogen peroxide removal, heat treatment and acid neutralization experiments (Figure 5). Protease treatment, the removal of hydrogen peroxide with catalase and heat treatment had no effect on the antifungal activity of the CFS (*p* > 0.05), which suggested that there were other antifungal substances in addition to enzymes, proteins and hydrogen peroxide. However, when the pH of the CFS was adjusted to six, the fungal growth inhibition was reduced significantly (*p* < 0.05). When the pH of the fermentation supernatant was adjusted to near neutral, the antibacterial activity of the treated fermentation supernatant decreased and was significantly lower than that of the untreated fermentation supernatant (*p* < 0.05), indicating that the acidic substances played an important role in the antibacterial activity of ZZUA493. In a previous report, the antifungal activity of the LAB fermentation supernatant, after the pH was adjusted to 7.0, was not different from that of the control group [32], which was consistent with our results. Guimaraes et al. reported similar results for *Lactobacillus plantarum* strains [25]. Therefore, it is speculated that the organic acids in the CFS of ZZUA493 play a key role in inhibiting fungi. 

**Figure 6:**
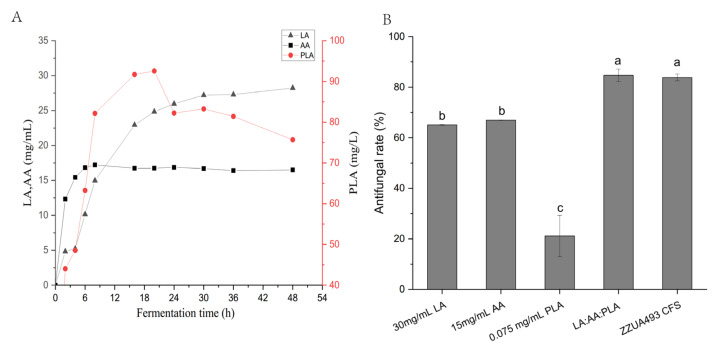
The organic acid production curve of ZZUA493 (**A**). The antifungal rate of LA, AA, PLA and their mixtures and CFS (**B**). Values with different superscript lowercase letters show significant differences among treatments (*p* < 0.05). LA, lactic acid; AA, acetic acid; PLA, phenyllactic acids. LA: AA: PLA, the mixtures of 30 mg/mL LA, 15 mg/mL AA and 0.075 mg/mL PLA; CFS, cell-free supernatant.

With the deepening of research, more and more scholars have begun to explore the exact substances of LAB that have antifungal activity. Corsetti A et al. reported that *Lactobacillus Sanfrancisco* CB1 from sourdough could inhibit *Fusarium*, *Penicillium*, *Aspergillus* and *Monilia* and the antibacterial substances were identified as formic acid, AA, propionic acid, butyric acid, n-valeric acid and hexanoic acid [14]. Fernandez A et al. reported that *Lactobacillus rhamnosus* A238 alone or in combination with *Bifidobacterium animalis subsp. lactis* A026 inhibited mold growth for at least 21 days at 6 °C; its inhibitory substances were LA, AA, PLA and 4-hydroxy-phenylenetic acid [33]. Prema P et al. found that the main antibacterial substance of *Lactobacillus plantarum* from silage was PLA [34]. Quattrini et al. found that *L. plantarum* strain ITEM 17215 showed a strong inhibitory ability towards fungal growth and was able to produce 1, 2-Dihydroxybenzene benzene, benzoic acid, P-hydroxyphenylenik acid and PLA, and the addition of phenylalanine and phenylpyruvate to the growth medium further increased the concentration of PLA [35]. PLA has the strongest antifungal activity compared with the same concentration of LA, hydroxyphenyllactic acid and indole LA [25]. In this study, HPLC was used to detect the contents of LA, AA and PLA in the supernatant of ZZUA493 after 48 h fermentation (Figure 6A). As can be seen from the figure, the contents of LA, AA and PLA tended to be stable after 48 h of fermentation, amounting to 28.5, 15.5 and 0.075 mg/mL, respectively. The antifungal activities of LA, AA and PLA were compared with those of the CFS fermented for 48 h by ZZUA493 alone or in mixture, and the results are shown in Figure 6B. For LA, when the concentration was 30mg/mL, the antifungal rate reached 65.06%. For AA, the antifungal rate was 66.94% when the concentration was 15 mg/mL, while, for PLA, the antifungal rate was 21.15% when the concentration was 0.075 mg/mL. When the three acids were mixed together, the antifungal rate was 84.74%, which was not significantly different from that of the CFS fermented for 48 h by ZZUA493. The results showed that LA, AA and PLA were the main antifungal substances in the CFS of ZZUA493.

**Figure 7:**
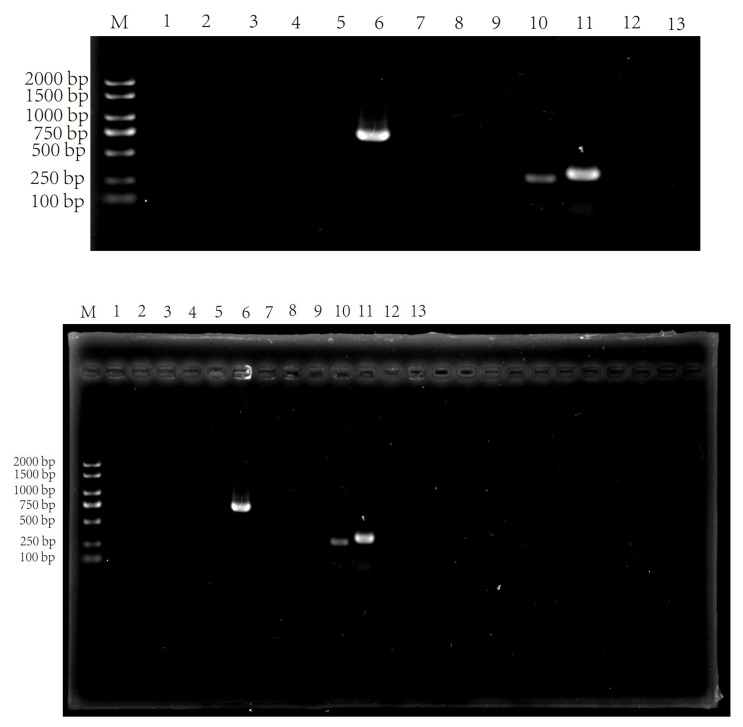
Agarose gel (1.5%) electrophoresis of the PCR products of bacteriocin-related genes. Lane numbers represent bacteriocin-related genes as follows: M. DL2000 plus DNA Marker; 1. *pln423*; 2. *plnQ*; 3. *plnN*; 4. *PlnNC81F*; 5. *plnXY*; 6. *plnW*; 7. *plnJK*; 8. *plnA*; 9. *plnEF*; 10. *plnS*; 11. *ped*; 12. *pln*; 13. *ent*.

Besides the acid produced by LAB, bacteriocin, as a safe and effective bacteriostatic substance, has an important prospective application. Bacteriocin is a kind of peptide or precursor peptide with antibacterial activity that is synthesized by bacteria through ribosomes after being stimulated by an external environment or growing to a certain concentration during their growth and metabolism [36]. Lavermicocca P et al. found that the growth of *A. niger* in bread produced by fermentation with *Lb. Plantarum* 21B that produces PLA was delayed by 7 days, and, in addition to organic acids and PLA, cyclic dipeptides also inhibit the growth of fungi to a certain extent, thus playing an important role in extending the shelf life of bread [31]. Saugar et al. found that the sequence of 11 amino acids located in the n-terminal α-helix region of cecropin derivatives was related to antifungal activity [37]. Lee et al. found that, after the fungi were treated with antimicrobial peptides, holes were formed in the membranes of the fungi [38]. With the continuous enrichment of bioinformation such as coding genes, the PCR rapid identification method for screening bacteriocin-producing LAB not only saves a lot of time and workload, but also significantly improves the accuracy [39]. Li et al. quickly detected class IIa bacteriocin-producing LAB with this method [27]. Macwana et al. discovered LAB producing three new bacteriocins based on the PCR method [40]. In order to verify the presence of bacteriocin in ZZUA493, the total DNA of ZZUA493 was used as the template and 13 pairs of bacteriocin-related gene-specific primers were used for PCR amplification. As shown in Figure 7, both *plnW*, *plnS* and *ped* could be amplified into a single band, which was consistent with the expected fragment size. The *plnW* gene encodes a membrane protein, the *plnS* gene is located on the transport operon and is responsible for bacteriocin secretion [41], and the *ped* gene is a gene related to Pediocin, which is widely studied at present [27]. The reports above show that these three genes play important roles in the process of bacteriocin synthesis. 

### 3.4. Effect of ZZUA493 on the Shelf-Life of CSB

**Figure 8:**
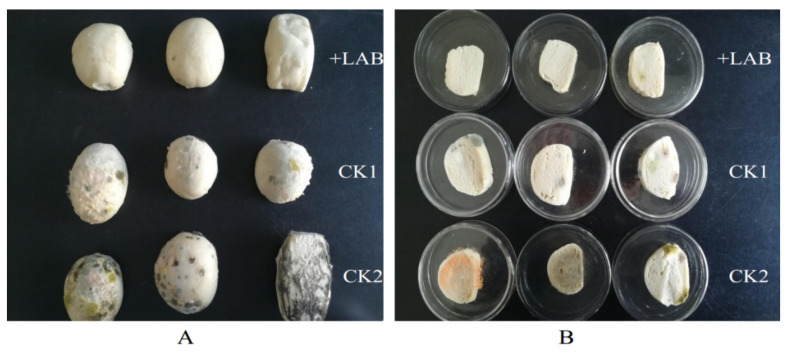
CSB in bag (**A**) and sliced (**B**) steamed bun placed for 6 days. CSB, Chinese steamed buns; LAB, lactic acid bacteria, Batter: 2mL ZZUA493 suspension + 100mL water + 100g flour fermented at 30 °C for 14 h; CK1, Batter: 2 mL water + 100 mL water + 100 g flour fermented at 30 °C for 14 h control; CK2, Batter: 2 mL water + 100 mL water + 100 g flour unfermented control.

Cereal-based fermented products are an important, low-cost source of human nutrition, because of their content of protein, carbohydrates, dietary fibers and trace elements [42]. CSB play an important role in Chinese people’s daily lives and provide an important dietary source of carbohydrates. Sourdough (i.e., lactic) fermentation can impart a desirable taste and nutritional properties and protects bread from fungal spoilage when LAB with antifungal activity are used, which can reduce the need for chemical preservatives [43]. ZZUA493 did not inhibit the growth of angel yeast in the dual-culture overlay assay method (Figure S2); therefore, a suspension of ZZUA493 was added to flour in order to prepare sourdough. After the sourdough fermentation, the pH was reduced to 4.6, while the pH of the control dough without ZZUA493 (CK) was 6.0. Adding LAB can impart an acidic taste to the dough and could also produce aromatic compounds, because of the proteolytic and lipolytic activities in LAB [44]. These substances contribute to the particular flavor of the sourdough. After steaming, our sourdough buns had a sour aroma, while the CK buns had an unpleasant smell. The sourdough buns had a soft mouthfeel, which was more palatable than the CK. In this experiment, compared with the CK group, the addition of acid dough composed of ZZUA493 and yeast significantly delayed the growth rate of the mold on the surface of the CSB (Figure 8), indicating that ZZUA493 has the potential to prolong the shelf life of CSB and then serve as a biological preservative.

## 4. Conclusions

In conclusion, we tested the antifungal activity of 359 strains of LAB in this study; among them, ZZUA493 had the highest activity against four different fungal species. There was good evidence that organic acids produced by ZZUA493 played an important role in fungal inhibition. The inclusion of sourdough fermentation with ZZUA493 in the preparation method for CSB approximately doubled their shelf life and improved both their flavor and texture. Therefore, ZZUA493 appears to have great potential as a natural, functional ingredient to markedly extend the shelf life of CSB.

## Figures and Tables

**Table 1:** The physiological and biochemical characteristics of ZZUA493.

Gram Stain	Shape	Fermentation Type	Growth at Temperature (°C)						Growth in NaCl (*w*/*v* %)		Gas from Glucose	Catalase
			4	10	20	30	40	50	3.0	6.5		
+	Rod	Homo	w	+	+	+	+	w	+	+	-	-
Growth at pH												
3.0	3.5	4.0	4.5	5.0	5.5	6.0	7.0	8.0	8.5	9.0	9.5	10
+	+	+	+	+	++	++	+	+	+	+	+	+

Notes: ++, Very good growth; +, Good growth; w, Weak growth; -, No growth.

## Data Availability

The ITS gene sequence of strains A, L, M and Q, used to support the findings of this study, have been deposited in the GenBank repository with accession numbers OM049201, OM049202, OM049222 and OM049221, respectively.

## Supplementary Materials

The following are available online at https://www.mdpi.com/article/10.3390/foods11020195/s1, Figure S1: Universal primers for four species of fungi PCR amplification product agarose gel electrophoresis, Table S1: Specific PCR primers for genes involved in bacteriocin biosynthesis, Table S2: Antibacterial activity of ZZUA493, Figure S2: Effect of ZZUA493 on Angel yeast.
