# Supplementary material for: Antifungal Activity of Lactobacillus plantarum ZZUA493 and Its Application to Extend the Shelf Life of Chinese Steamed Buns
Source: Foods. 2022 Jan 12;11(2):195. doi: 10.3390/foods11020195 (PMC8775031; doi:10.3390/foods11020195)
Supplement: Supplementary file 1 [file foods-11-00195-s001.zip › foods-1505546-supplementary.pdf]

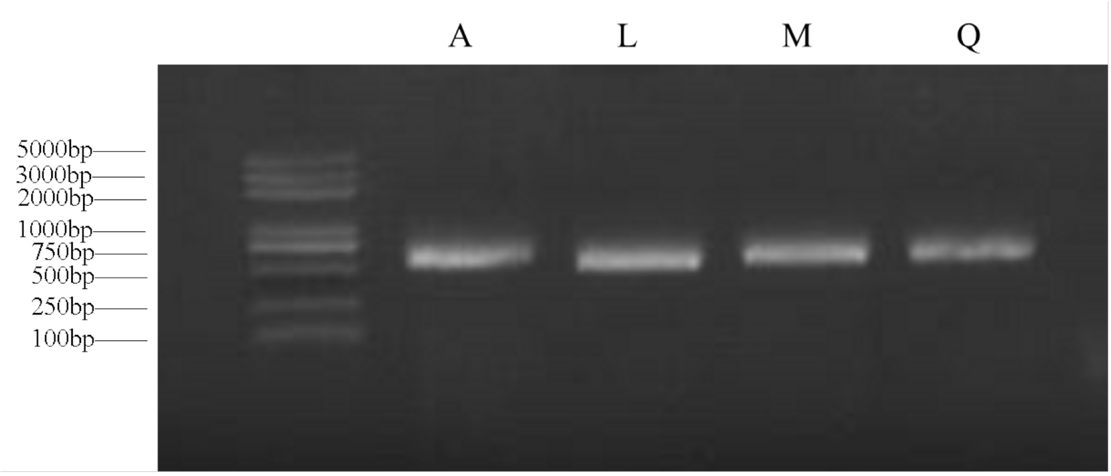

Figure S1: Universal primers for four species of fungi PCR amplification product agarose gel electrophoresis

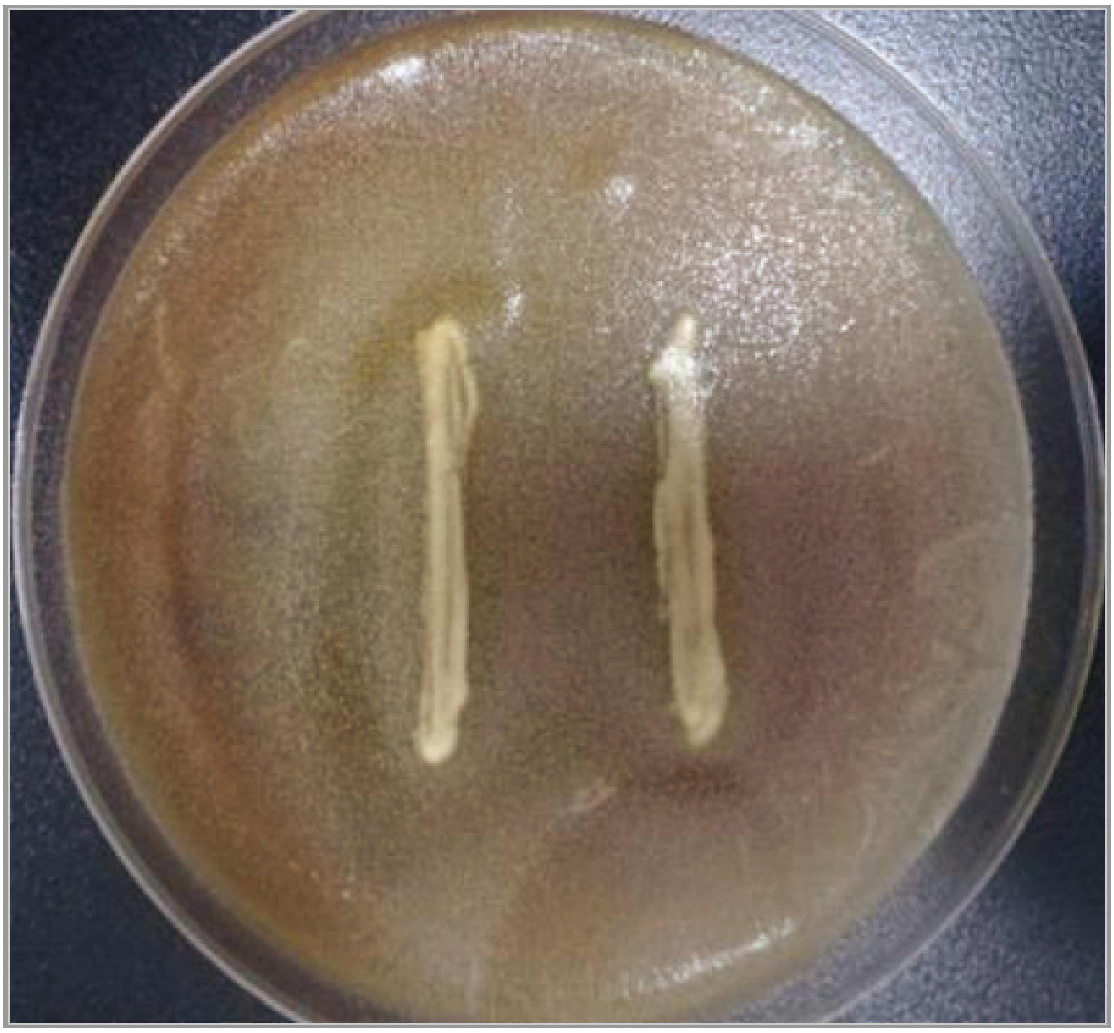

Figure S2: Effect of ZZUA493 on Angel yeast.

Table S1. Specific PCR primers for genes involved in bacteriocin biosynthesis

Target gene	Sequence(5'→3')		Annealing temperature (°C)	Length
	Forward primer	Reverse primer		
pln423	TATGATGAAAAAAATTGAAAAAT	CCAAAGATAATCCCCCCCCAT	50	197
plnQ	TGAAATCCTACAATATGAAATTGAACCGCGA	TTATTTTCTCTTACTTGTAAAGGCTCTCAA	55	188
plnN	ATTGCCGGGTTAGGTATCG	CCTAAACCATGCCATGCAC	51.9	146
plnNC81F	TTGGCGGAAAAACAAAGACT	TCAGCATGTCATTTCACCATC	52.5	114
plnXY	ATTCAGCGATTAGCATTG	GGAGCCATAAACTCTTCTT	52.9	286
plnW	ATGTTACAGAAGAATTTACGGT	TTAGCTAGGAACCAACCAG	54.8	686
plnJK	GCCACAAAGAGCACTAACA	ATGACTGTGAACAAAATGA	54.8	427
plnA	ATGAAAATTCAAATTAAAGG	TTACCATCCCCATTTTTTA	55	146
plnEF	TGATGGCTTGAACTATCCGTG	CATACAAGGGGGATTATTT	58.3	385
plnS	ATGGCACACTCAAATAAAC	TCAACAATAATGAGCACGA	58.3	299
ped	GGTAAGGCTACCACTTGCAT	CTACTAACGCTTGGCTGGCA	55	332
pln	CCAGCAGTTCTTCCAATTTCA	CAGGTTGCCGCAAAAAAAG	55	608
ent	GGGTACCACTCATAGTGGAA	CCAGCAGTTCTTCCAATTTCA	55	412

Table S2. Antibacterial activity of ZZUA493

strains	Antibacterial activity					
	***S.aureus* ATCC 29213**	***M.luteus* ATCC 4698**	***B.subtilis* ATCC 6633**	***P.aeruginosa* ATCC 27853**	***E.coli* ATCC 30105**	***L.monocytogenes***
ZZUA493	++	+++	+++	++	++	+++

Notes: Diameter of inhibition zone: -, no inhibition; +, 10.00-15.00 mm; ++, 16.00-20.00 mm; +++, > 20.00 mm. The inhibition zone contains the external diameter of the cup (7.80 mm).
